# Current status of indwelling urinary catheter utilization and catheter-associated urinary tract infection throughout hospital wards in Korea: A multicenter prospective observational study

**DOI:** 10.1371/journal.pone.0185369

**Published:** 2017-10-09

**Authors:** Bongyoung Kim, Hyunjoo Pai, Won Suk Choi, Yeonjae Kim, Ki Tae Kweon, Hyun Ah Kim, Seong Yeol Ryu, Seong-heon Wie, Jieun Kim

**Affiliations:** 1 Department of Internal Medicine, Eulji University College of Medicine, Daejeon, Korea; 2 Department of Internal Medicine, Hanyang University College of Medicine, Seoul, Korea; 3 Department of Internal Medicine, Korea University College of Medicine, Ansan, Korea; 4 Department of Infectious Disease, National Medical Center, Seoul, Korea; 5 Department of Infectious Disease, Patima Hospital, Daegu, Korea; 6 Department of Internal Medicine, Gyemyeong University College of Medicine, Daegu, Korea; 7 Department of Internal Medicine, St. Vincent Hospital, Suwon, Korea; University of Calgary, CANADA

## Abstract

To evaluate the frequency and appropriateness of indwelling urinary catheters (IUC) use and the incidence of catheter-associated urinary tract infections (CA-UTI), and explore the risk factors for CA-UTI in hospitals as a whole, we conducted a study. This study was divided into two parts; a point-prevalence study on Dec 12^th^ 2012 and a prospective cohort study from Dec 13^th^ 2012 to Jan 9^th^ 2013 were performed in six hospitals in Korea. All hospitalized patients with newly-placed IUCs were enrolled and monitored weekly for 28 days after IUC placement. In the point-prevalence study, the IUCs were present in median 14.9/100 hospitalized patients (1Q 14, 3Q 16) across the six hospitals. In the prospective cohort study, the median IUC-days per patient was 5 (1Q 3, 3Q 10) and the median CA-UTI prevalence per 1,000 catheter days was 1.9 (1Q 0.7, 3Q 3.8) with significant inter-hospital variation. The proportion of patients with inappropriate IUC maintenance increased with number of IUC-days (8.5% on day 7, 9.4% on day 14, 16.3% on day 21, and 23.1% on day 28). Urinary output monitoring (23/36, 63.9%) was the most common indication for inappropriate use after 1 week of ICU placement. In multivariate analysis, IUC-days was significantly associated with the development of CA-UTI (odds ratio 1.122, 95% confidence interval 1.074–1.173, *P*< 0.001). IUC-days and CA-UTI rates vary between hospitals. IUC-days is a risk factor for CA-UTI, and is correlated with inappropriate use.

## Introduction

Catheter-associated urinary tract infection (CA-UTI) is the most common nosocomial infection, accounting for up to 10–70% of all nosocomial infections especially in intensive care unit (ICU) setting [1–3]. Approximately 3–7% of catheterized patients acquire a new infectious organism per day, and the prevalence of bacteriuria approaches 100% by 30 days after catheterization [4]. Symptomatic CA-UTI develops in 24% of patients with bacteriuria, and bacteremia from CA-UTI develops in 3.6% of patients [5]. These complications of indwelling urinary catheters (IUC) are associated with considerable morbidity, prolonged hospitalization, and increased health care expenditure [6]. In one study, CA-UTI patients incurred a mean of $589 (median $356) of extra costs per patient for diagnostic tests and medication [7]. Hence, strategies to prevent CA-UTI have been emphasized in many countries and hospitals.

The current guideline for prevention of CA-UTI recommends to minimize duration for catheterization and maintain sterile technique for insertion and keep closed drainage system [8]. Accordingly, each physician should insert catheters only for appropriate indications and leave in place only as long as needed [9]. Implement records of indication for insertion, date of catheter insertion, and daily presence of a catheter maintenance also prevent CA-UTI. Furthermore, a systematic review found that the CA-UTI rate was reduced by 52% with use of a reminder or stop order which prompt IUC removal [10].

Estimating the current status of IUC utilization and the burden of CA-UTI is indispensable for developing and evaluating strategies for its prevention and control of CA-UTI. Identifying risk factors is also important for identifying priority group for intervention. Most studies of CA-UTI have focused on the intensive care unit (ICU) population. In Korea, data on CA-UTI acquired in ICUs has been collected through the Korean National healthcare-associated Infections Surveillance System (KONIS) since 2006 [2]. However, there have been few studies of IUC use, CA-UTI rates and risk factors for CA-UTI in the general wards of Korean hospitals.

This study aimed to assess the frequency and appropriateness of IUC use and the incidence of CA-UTI, and to explore risk factors associated with CA-UTI among patients with IUCs throughout the wards of hospitals.

## Materials and methods

### Study design and setting

Six hospitals with 543–791 beds participated in the study. They were: Hanyang University Seoul Hospital (758 beds), Korea University Ansan Hospital (543 beds), Daegu Patima Hospital (657 beds), Keimyung University Dongsan Hospital (783 beds), St. Vincent's Hospital (791 beds), and Hanyang University Guri Hospital (578 beds).

### Point-prevalence study

On December 12^th^, 2012, researchers in each hospital collected information on the total number of hospitalized patients, the number of patients with IUCs and the number with CA-UTI in all the wards of each hospital. There was no missing data for point-prevalence study.

### Prospective cohort study

#### Patient population

Between December 13th, 2012 and January 9th, 2013, all hospitalized patients with newly- placed IUCs were enrolled. Patients were excluded if they: (1) were under 18 years old, (2) died, were discharged, or were transferred to other medical institutions within 48 hours of IUC placement, and (3) received the IUC within 48 hours of the removal of a previous UC.

#### Data collection

The following information was collected at enrollment: demographic features (age and gender), use of other instruments (central venous catheter, nasogastric tube, endotracheal tube, or ventilator), operation history location (brain, spine, knee, stomach, or colon) within the previous month, and underlying co-morbidities included hypertension, ischemic heart disease, congestive heart disease, asthma, chronic obstructive pulmonary disease, hemodialysis, liver cirrhosis, cerebrovascular accident, malignancy, diabetes with/without complications, and status of chronic kidney disease (mild/moderate/severe).

Thereafter follow-up monitoring was conducted weekly for day 28 (days 7, 14, 21, and 28 from the day of IUC placement). We assessed whether the IUCs were placed and maintained appropriately, and checked for the development of CA-UTI. If patients were discharged or transferred to other hospital with maintained IUCs, we regarded as dropped out of observation. Remained patients with IUCs were categorized with maintained or removed IUCs group. The date of IUC removal was collected in order to calculate IUC-days.

The purpose of IUC placement was recorded by healthcare personnel on the day of insertion and every follow-up monitoring day; multiple answers were allowed. Appropriate indications for IUC placement were: to relieve acute urinary retention, to measure urinary output accurately in critically-ill patient, to manage perioperative conditions, to assist in healing of open sacral or perineal wounds in incontinent patient, to improve comfort in end-of-life care, and to support prolonged immobilization [8]. The adequacy of catheter maintenance was evaluated by an infectious diseases (ID) specialist: use was considered "inappropriate" when the ID specialist considered it was not justified by any of the above criteria.

When CA-UTI was diagnosed during follow-up, we collected information about the causative organism. If there were more than two episodes of CA-UTI in a single patient, only the first episode was included.

### Definitions related to IUC and CA-UTI

Urinary catheterization was defined as insertion of a Foley catheter through the urethra. The urinary catheter utilization ratio was defined as the number of urinary catheter days divided by the number of patient days. Point-prevalence was defined as the frequency of all current events on December 12^th^, 2012 [11]. In terms of duration of IUC placement, we counted ≥28 days of IUC use as 28 days.

CA-UTI was defined as follows among all patients with IUCs, including those whose urinary catheters were removed within 48 hours: presence of at least one of the following signs or symptoms that could not be explained by other causes (fever ≥38.0°C, urgency, frequency, dysuria, suprapubic tenderness, and costovertebral angle pain or tenderness) together with a positive urine culture (≥ 10^5^ CFUs/ml) with ≤ 2 bacterial species or at least one positive outcome in the dipstick test, pyuria, and gram stain. Patients with a positive urine culture on the day of IUC placement were excluded.

### Statistical analysis

Categorical variables were analyzed by the Chi-square test or Fisher's exact test. Continuous variables were analyzed by independent *t*-tests or the Mann-Whitney U-test. A logistic regression analysis was performed to evaluate the effect of independent variables on risk. A *P*-value of <0.05 in a two-tailed test was considered to be statistically significant. To assess inter-hospital differences in urinary catheter days, we used the Kruskal-Wallis test with the Bonferroni correction, and considered a *P*-value of <0.0083 significant. All analyses were performed using SPSS Statistics version 21.0 (IBM Corporation, Armonk, NY).

### Ethics statement

The study protocol was approved by the institutional review boards of Hanyang University Guri Hospital (IRB number: 2012-11-085), and the requirement for written informed consent from patients was waived.

## Results

### The point-prevalence study

IUCs were present in 14.9% (576/3,870) of hospitalized patients [median 14.9/100 patients, (1Q 14, 3Q 16)] on the day of examination. The point-prevalence of CA-UTI was 0.39 (1Q 0.23, 3Q 1.14) per 100 admissions or 2.39 (1Q 1.51, 3Q 8.35) per 100 patients with IUCs (Table 1).

**Table 1:** Point-prevalence of indwelling urinary catheter, catheter utilization ratio and urinary tract infections.

Hospital	Total patients	Patients with IUCs	CA-UTI	Point prevalence		
				IUC utilization ratio*	CA-UTI	
					per 100 admission	per 100 patients with IUCs
A	862	129	2	0.15	0.23	1.55
B	553	71	8	0.13	1.45	11.27
C	680	95	7	0.14	1.03	7.37
D	473	73	1	0.15	0.21	1.37
E	613	87	2	0.14	0.33	2.3
F	689	121	3	0.18	0.44	2.48
Total	3,870	576	23			
Median (1Q, 3Q)	647 (533, 732)	91 (73, 123)	3 (2, 7)	0.15 (0.14, 0.16)	0.39 (0.23, 1.14)	2.39 (1.51, 8.35)

IUC, indwelling urinary catheter; CA-UTI, catheter-associated urinary tract infections

* Patients with IUCs/total patient

### The prospective cohort study

#### Patient characteristics

A total of 1,298 patients were screened during the 4-week study. Fifty patients were excluded for the following reasons: under 18 years of age (14 patients), and discharged within 48 hours of IUC placement (36 patients). In the end 1,248 patients were enrolled in the study.

The median age of the patients was 64 years (1Q 50, 3Q 74), and 57.4% were female. The median observation period was 13 days (1Q 7, 3Q 22) and the median duration of IUC use was 5 days (1Q 3, 3Q 10).

Table 2 shows inter-hospital differences in IUC use and CA-UTI incidence. There were 9,591 total catheter days and a median of 1,607 catheter days (1Q 1,391, 3Q 1,840). Catheter days per patient differed significantly between hospitals (Kruskal-Wallis test; *P*<0.001). Patients in hospitals A and C had significantly more catheter days than those in the other hospitals (*P*-value < 0.0083; *P*-value for multiple comparison between A and B <0.001, between A and D 0.028, between A and E <0.001, between A and F 0.005, between B and C <0.001, between B and D >0.99, between B and E >0.99 between B and F >0.99, between C and D 0.014, between C and E <0.001, between C and F 0.002, between D and E 0.876, between D and F >0.99, and between E and F 0.726.

**Table 2:** Inter-hospital differences in indwelling urinary catheter use and evaluation and prevalence of catheter-associated urinary tract infections.

Hospital	IUC utilization			Frequency of urine culture		Prevalence of CA-UTI	
	Total patients (%)	IUC-days, total	IUC-days, per patient, median (1Q, 3Q)	Number of cultures (%)	Number of cultures/1,000 IUC-days	Number of CA-UTI (%)	CA-UTI/1,000 IUC-days
A	154 (12.3)	1,527	7 (5, 13)	66 (23.2)	43.2	1 (4.2)	0.7
B	248 (19.9)	1,686	4 (2, 8)	56 (19.6)	33.2	5 (20.8)	3
C	197 (15.8)	1,952	7 (4, 13)	72 (25.3)	36.9	12 (50.0)	6.1
D	156 (12.5)	1,152	5 (3, 9.5)	9 (3.2)	7.8	2 (8.3)	1.7
E	245 (19.6)	1,471	3 (2, 8)	46 (16.1)	31.3	2 (8.3)	1.4
F	248 (19.9)	1,803	4 (2, 9)	36 (12.6)	20	1 (4.2)	0.6
Total	1,248 (100)	9,591		285 (100)		23 (100)	
Median (1Q, 3Q)	221 (156, 248)	1,607 (1,391, 1,840)	5 (3, 10)	51 (29, 68)	32.3 (17.0, 38.5)	2 (1, 6.8)	1.6 (0.7, 3.8)

IUC, indwelling urinary catheter; CA-UTI, catheter-associated urinary tract infections

#### Placement, maintenance, and removal of IUCs

**Fig 1:**
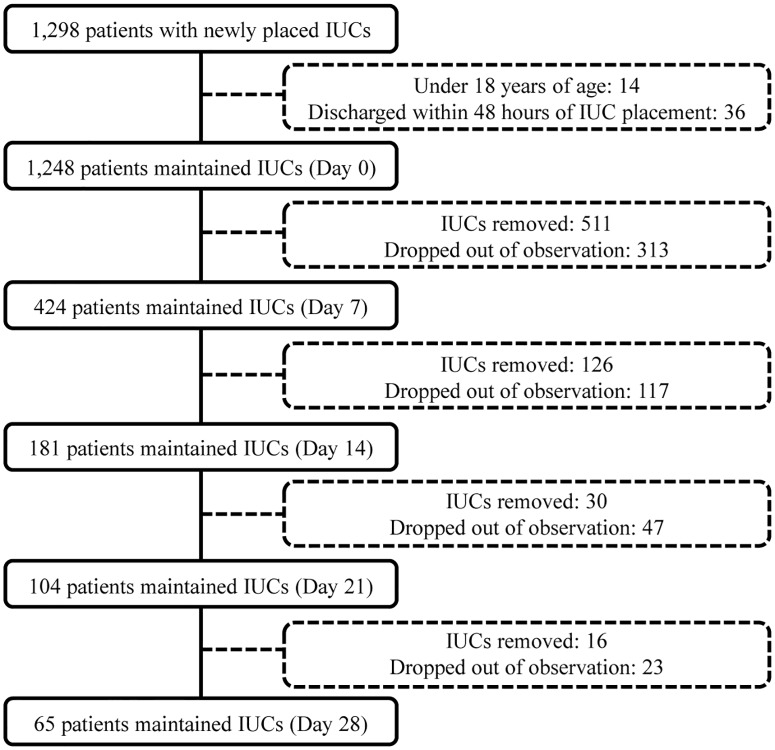
A flow diagram showing status of indwelling urinary catheters. From the placement of urinary catheter through day 28 of follow-up monitoring, maintenance and removal of indwelling urinary catheter was traced weekly.

After 1 week of IUC placement, 511 patients (511/1,248, 40.9%) had had their IUC removed and 313 patients (313/1,248, 25.1%) had dropped out of observation. Of the remaining 424 patients (424/1,248, 34.0%), 36 (36/424, 8.5%) had no adequate indication for use of an IUC. After 2 weeks, 126 patients (126/424, 29.7%) had had their IUC removed, 117 (117/424, 27.6%) had dropped out, and 181 (181/424, 42.7%) remained, with 9.4% inappropriate use. After 3 weeks, 30 patients (30/181, 16.6%) had had their IUC removed, 47 (47/181, 26.0%) had dropped out, and 104 (104/181, 57.5%) remained, with 16.3% of inappropriate use. After 4 weeks, 16 patients (16/104, 15.4%) had had their IUC removed, 23 (23/104, 22.1%) had dropped out, and 65 (65/104, 62.5%) remained, with 23.1% of inappropriate use (Fig 1).

#### Indications for IUC use recorded by healthcare personnel

The most common indication for initial IUC use was perioperative care (594, 47.6%), followed by close monitoring of urinary output (590, 47.3%) and relief of urinary retention (196, 13.1%) (Table 3). Over the period that IUCs were monitored, the proportion of urinary catheters used for perioperative care decreased (10.8%, day 7; 5.5%, day 14; 2.9%, day 21; 4.6%, day 28) and the proportion used for close monitoring of urinary output increased (64.2%, day7; 70.2%, day 14; 72.1%, day 21; 76.9%, day 28).

**Table 3:** Indications for indwelling urinary catheter (IUC) use and adequacy of IUC maintenance during the period of weekly monitoring.

Indication	IUC-day median (1Q, 3Q)	Days from IUC placement N (%)								
		the day of placement	Day 7		Day 14		Day 21		Day 28	
			Total use	Inapp. use	Total use	Inapp. use	Total use	Inapp. use	Total use	Inapp. use
Acute urinary retention	8 (5,14.8)	196 (15.7)	111 (26.2)	6 (16.7)	43 (23.8)	1 (5.9)	23 (22.1)	2 (11.8)	12 (18.5)	0 (0.0)
Urinary output monitoring	7 (4,13)	590 (47.3)	272 (64.2)	23 (63.9)	127 (70.2)	13 (76.5)	75 (72.1)	14 (82.4)	50 (76.9)	13 (86.7)
Open sacral or perineal wound	10 (5,24)	11 (0.9)	12 (2.8)	4 (11.1)	6 (3.3)	1 (5.9)	6 (5.8)	1 (5.9)	3 (4.6)	1 (6.7)
Comfort for end of life	6 (3,12.8)	36 (2.9)	13 (3.1)	3 (8.3)	8 (4.4)	0 (0.0)	4 (3.8)	0 (0.0)	2 (3.1)	0 (0.0)
Perioperative care	3 (2,5.3)	594 (47.6)	46 (10.8)	0 (0.0)	10 (5.5)	1 (5.9)	3 (2.9)	0 (0.0)	3 (4.6)	1 (6.7)
Other	6 (4,10)	67 (5.4)	34 (8.0)	2 (5.6)	18 (9.9)	0 (0.0)	10 (9.6)	1 (5.9)	6 (9.2)	0 (0.0)
Total	5 (3, 10)	1248	424	36	181	17	104	17	65	15

Inapp., Inappropriate; IUC, indwelling urinary catheter

Multiple answers were allowed

#### Adequacy of IUC maintenance

The greater the number of IUC-days, the higher was the proportion of patients using IUCs without appropriate indications (8.5% (36/424), day 7; 9.4% (17/181), day 14; 16.3% (17/104), day 21; 23.1% (15/65), day 28. For the 36 inappropriate IUCs used after 1 week of IUC placement, urinary output monitoring (23/36, 63.9%) was most common indication, followed by acute urinary retention (6/36, 16.7%) (Table 3). The proportion of IUCs maintained for urinary output monitoring as inappropriate indication increased over the period of monitoring (76.5%, day 14; 82.4%, day 21; 86.7%, day 28).

#### Inter-hospital differences in median IUC-days according to operation types

To minimize the inevitable biasing of IUC-days according to operation type, we performed a sub-analysis of IUC-days by common operations. Brain operations were performed in 68 patients and the median IUC-days was 7 (1Q 4, 3Q 17.8). There were no inter-hospital differences in IUC-days associated with brain operations (*P* = 0.053 by Kruskal-Wallis test). Spine operations were performed on 63 patients and the median IUC-days was 4 (1Q 2, 3Q 7) with no significant inter-hospital differences (*P* = 0.296). Knee operations were performed in 40 patients and the median IUC-days was 3.5 (1Q 3, 3Q 5) with significant inter-hospital differences (*P*<0.001). There were significant differences in IUC-days between hospitals C and E, and hospitals D and E, after the Bonferroni correction (*P*<0.001 and <0.001, respectively). Colorectal surgery was performed in 29 patients and the median IUC-days was 3 (1Q 2, 3Q 6) with significant inter-hospital differences (*P* = 0.049). There were significant differences in catheter days between hospital D and F, and hospital E and F, (*P* = 0.036 and 0.006, respectively). Only the *P*-value for hospitals E and F was significantly different after the Bonferroni correction. Stomach operations were performed in 26 patients and the median IUC-days was 3 (1Q 2.8, 3Q 6) with significant inter-hospital differences (*P* = 0.027). There were significant differences in catheter days between hospital B and D, and hospital D and E, (*P* = 0.006 and 0.029). Only the *P*-value for hospitals B and D was significantly different after the Bonferroni correction.

#### CA-UTI

A total of 285 urine cultures were set up, and the median number of urine cultures per 1,000 catheter days was 32.3 (1Q 17.0, 3Q 38.5). A total of 25 pathogens were identified by urine culture from 23 patients with CA-UTI. *Enterococcus* spp. was the leading causative organism (8/25, 32%), followed by *Escherichia coli* (7/25, 28%) (Table 4). The median prevalence of CA-UTI per 1,000 catheter days was 1.6 (1Q 0.7, 3Q 3.8).

**Table 4:** Causative organisms of catheter-associated urinary tract infection.

Pathogens	Number (%)
*Acinetobacter spp*.	2 (8)
*Candida spp*.	4 (16)
*Enterococcus spp*.	8 (32)
*Escherichia coli*	7 (28)
*Proteus mirabilis*	1 (4)
*Pseudomonas spp*.	2 (8)
*Staphylococcus spp*.	1 (4)
Total	25^a^ (100)

^a^ Total number of identified organisms from 23 patients

The clinical characteristics of patients with CA-UTI are compared with those of patients without CA-UTI in Table 5. The median age of the CA-UTI group was 69 (1Q 56, 3Q 74), and that of the non-CA-UTI group was 64 (1Q 50, 3Q 74.8) (*P* = 0.194). Male gender was more common in the CA-UTI group (62.5% vs. 42.4%, *P* = 0.049). Among underlying diseases, hypertension was more frequent in the CA-UTI group (65.2% vs. 42.8%, *P* = 0.037), but there were no significant differences for other parameters. As for the use of additional equipment, the CA-UTI group used ventilators more frequently than the non-CA-UTI group (21.7% vs. 6.5%, *P* = 0.008). There were no significant differences in the use of central venous catheters, nasogastric tubes and endotracheal tubes. The non-CA-UTI group underwent more operations within a month before enrollment than the CA-UTI group (52.3% vs. 30.4%, *P* = 0.044). Median IUC-days was significantly longer in the CA-UTI group than the non-CA-UTI group [18 days (1Q 1, 3Q 28) vs. 5 days (1Q 3, 3Q 9), *P*< 0.001]. Inappropriate use of IUCs on days 7, 14, 21, and 28 was not correlated with the development of CA-UTI (*P* = 0.709, >0.99, 0.163, and >0.99, respectively). In multivariate logistic regression analysis, only IUC-days was significantly associated with CA-UTI (Odd ratio 1.127, 95% confidence interval 1.077–1.180, *P*< 0.001).

**Table 5:** Clinical characteristics of catheter-associated urinary tract infection (CA-UTI) patients and non-CA-UTI patients.

	Total (n = 1248)	CA-UTI (n = 23)	Non-CA-UTI (n = 1225)	simple Odd ratio	*P*-value	multiple Odd ratio	*P*-value
Demographic data							
Age, median (1Q, 3Q)	64 (50, 74)	69 (56, 74)	64 (50, 74.5)	1.019 (0.992, 1.047)	0.161	0.997 (0.965, 1.029)	0.839
Male sex (%)	532 (42.6)	14 (60.9)	520 (42.4)	0.474 (0.204, 1.104)	0.083	1.767 (0.728, 4.291)	0.208
Underlying disease (%)							
Hypertension	539 (43.2)	15 (65.2)	524 (42.8)	2.508 (1.056, 5.960)	0.037	2.199 (0.842, 5.747)	0.108
Ischemic heart disease	56 (4.5)	1 (4.3)	55 (4.5)	0.967 (0.128, 7.305)	0.974		
Congestive heart failure	47 (3.8)	1 (4.3)	46 (3.8)	1.165 (0.154, 8.831)	0.883		
Asthma	21 (1.7)	1 (4.3)	20 (1.6)	2.739 (0.352, 21.319)	0.336		
COPD	22 (1.8)	0 (0.0)	22 (1.8)				
Hemodialysis	10 (0.8)	0 (0.0)	10 (0.8)				
DM	287 (23.0)	6 (26.1)	281 (22.9)	1.186 (0.463, 3.036)	0.723		
Utilization of other instruments (%)							
Central venous catheter	232 (18.6)	3 (13.0)	227 (18.5)	0.659 (0.194, 2.238)	0.504		
Nasogastric tube	188 (15.1)	6 (26.1)	182 (14.9)	2.023 (0.787, 5.198)	0.144		
Endotracheal tube	236 (18.9)	7 (30.4)	229 (18.7)	1.903 (0.774, 4.679)	0.161		
Ventilator	85 (6.8)	5 (21.7)	80 (6.5)	3.976 (1.439, 10.986)	0.008	1.791 (0.607, 5.289)	0.291
Operation history ^a^	648 (51.9)	7 (30.4)	641 (52.3)	0.399 (0.163, 0.976)	0.044	0.915 (0.346, 2.422)	0.858
IUC-days, median (1Q, 3Q)	5 (3, 10)	18 (11, 28)	5 (3, 9)	1.138 (1.090, 1.188)	<0.001	1.127 (1.077, 1.180)	<0.001

CA-UTI, catheter-associated urinary tract infections; COPD, chronic obstructive pulmonary disease; DM, diabetes mellitus; IUC, indwelling urinary catheter

^a^ Within one month before the day of enrollment

## Discussion

The purpose of this multicenter study was to examine the frequency and adequacy of IUC use, to identify reasons for catheter maintenance, and to assess the extent and risk of hospital-acquired CA-UTI associated with IUCs in hospital wards as a whole.

In a previous study by Lewis et al., the IUC utilization ratio was 0.83 in ICUs, 0.21 in non-ICUs, and 0.24 overall [12]. The incidence rate of CA-UTI per 1,000 catheter days was 1.21 throughout hospitals. Even though the IUC utilization ratio was lower in non-ICUs than ICUs, the incidence rates of CA-UTI were similar (1.31 and 1.33 per 1,000 catheter days in non-ICUs and ICUs, respectively). In this study, the IUC utilization ratio in all hospital wards was 0.15 and the value for ICUs given in the previous KONIS study by Lewis et al was 0.84 [2]. We performed a point-prevalence study to calculate the utilization ratio, and others have performed prospective surveillance studies. Therefore direct comparison with previous studies such as the KONIS may have limitations due to difference of study design. However, our study yielded findings resembling those of the Lewis study, in which CA-UTI prevalence per 1,000 catheter days was 2.6 in hospitals overall and 1.2 in ICUs. In other words, non-ICU patients use IUCs less than ICU patients, but CA-UTI occurs more frequently in non-ICU patients. These findings point to a need to monitor the adequacy of IUC use throughout hospital wards to lower the rate of CA-UTI.

Median IUC-days varied significantly among the participating hospitals in this study. The incidence of CA-UTI also varied: it was approximately 10 times more frequent in hospital C (6.1/1,000 catheter days) than in hospital F (0.6/1,000 catheter days). Even though we compared IUC-days by type of operation to minimize bias from patients’ characteristics, significant inter-hospital differences were noted. Thus, it is important to ensure the adequate use of IUCs and to implement infection controls against CA-UTI throughout hospital wards.

Increased length of IUC stay is a well-known risk factor for CA-UTI [6, 13]. Apisarnthanarak et al. demonstrated that patients who remained in IUCs inappropriately for prolonged times had a higher probability of developing CA-UTI [14], which prolonged hospitalization and increased costs. In the present study, the frequency of inappropriate use was 8.5% on day 7, lower than in previous studies (21–54%) [14, 15]. The frequency of inappropriate use increased with time to 23.1% at the end of the study period. We need to be aware that the greater the number of IUC-days, the higher the rates of inappropriate use of IUCs.

Reasons of inappropriate IUC use have varied between studies. Elpern et al. demonstrated that close monitoring for urinary output, no clear indication, and urinary incontinence were major indication for inappropriate use [16]. Admission to the medical ICU, non-ambulatory functional status, female gender, older age, and not having had surgery were independently associated with inappropriate use [14, 17]. In many countries and hospitals, medical staff including physicians are often unaware of the placement of IUCs in their patients [13, 15, 18]. This leads to prolonged installation of IUCs that are clinically unnecessary. These findings indicate that a large proportion of inappropriate and prolonged IUC use is preventable by careful monitoring. In this study, the most common reason for inappropriate IUC use was close monitoring of urinary output. Even though the number of instances of inappropriate use decreased with time (from 23 to 13), the proportion of instances installed for close monitoring of urinary output increased (from 63.9% to 86.7%). We suggest that healthcare personnel should be aware of IUCs that have been in place for more than a week to monitor urinary output, so as to prevent inappropriate use. Furthermore, strategies to enhance each medical staff’s adherence to guidelines of CA-UTI prevention are also necessary. Some studies showed promising strategies. Gokula et al. increased appropriate use of IUC from 37% to 51% in emergency room by using combined educational intervention and an indication checklist [19]. Other study showed that nurse-led multidisciplinary rounds were effective to reduce the unnecessary IUC use [20]. In addition, reminder or stop order was also helpful to reduce CA-UTI incidence [10].

There are several limitations to this study. First, we only included university hospitals. In Korea, there are a total of 3,472 hospitals and 2.3% (82/3,472) comprises university hospitals. Therefore, our results may not be generalizable to other types of hospital. Moreover, even though the participating hospitals had similar numbers of beds, the patients’ characteristics may have differed, which could have led to divergent results for urinary catheter management as well as CA-UTI rates. Second, we did not collect data on whether patients were hospitalized in ICUs or non-ICU wards. Therefore, we could not assess differences of CA-UTI incidence between ICUs and non-ICUs. Finally, the adequacy of IUC use was decided by researchers in the individual hospitals, and we cannot exclude the possibility of inter-researcher differences in making this decision.

This study showed that the overall incidence CA-UTI in hospitals including non-ICU wards was higher than in ICUs and the duration of IUC use, and CA-UTI rates, varied between hospitals. The main risk factor for CA-UTI was prolonged IUC use, which correlated with inappropriate use. All medical staff should be advised to be alert to inappropriate IUC use in order to prevent CA-UTI. Introducing tools that can be easily applied to promote appropriate management of IUCs and prevent and control CA-UTI in Korean hospitals would be a good strategy to enhance medical staff’s awareness. Further researches are required in the future.

## Supporting information

S1 File CA-UTI multicenter-dataset.sav. This file included raw data of this study except personal and potentially identifying participant data.
(SAV)
